# Interpretable, internally validated prognostic modelling of cumulative clinical pregnancy in women with diminished ovarian reserve

**DOI:** 10.1186/s12911-026-03709-5

**Published:** 2026-07-18

**Authors:** Huimei Wu, Qianyi Huang, Yu Long, Li Jiang, Lidan Liu

**Affiliations:** 1 Reproductive Medical Center, The First Affiliated Hospital of Guangxi Medical University, Nanning, China; 2 Department of Obstetrics, The First Affiliated Hospital of Guangxi Medical University, Nanning, China

**Keywords:** Diminished ovarian reserve, Cumulative clinical pregnancy, Risk stratification, Interpretable machine learning, SHAP

## Abstract

**Objective:**

To develop and internally validate an interpretable prognostic model for cumulative clinical pregnancy in women with diminished ovarian reserve (DOR), and to explore, as a hypothesis-generating secondary aim, whether protocol-associated pregnancy rates differ across model-defined baseline-prognosis risk strata.

**Design:**

Retrospective cohort study.

**Setting:**

A single tertiary reproductive medicine center.

**Patients:**

1,251 oocyte-retrieval cycles in women with DOR (AMH ≤ 1.1 ng/mL) at a single tertiary centre, January 2019–July 2024. Each record is one retrieval cycle; no patient identifier was available.

**Methods:**

From 37 candidate predictors, random-forest selection (training set only) retained eight; six algorithms were compared for discrimination (AUC, DeLong), calibration (slope, intercept, Brier) and clinical utility (decision-curve analysis), with SHAP for interpretability. Discrimination was further assessed by out-of-time validation (train ≤2022, test 2023–2024). Within model-defined risk strata, protocols were compared (chi-square/Fisher; bootstrap CIs; FDR and Bonferroni correction), with a pre-treatment-variable-only sensitivity analysis.

**Results:**

A parsimonious logistic regression gave the highest test AUC (0.739, 95% CI 0.688–0.790; Brier 0.200), not significantly better than more complex algorithms (DeLong p>0.10), and held up on out-of-time validation (AUC 0.726). The five most influential predictors (SHAP) were embryo quality, female age, male age, estradiol and AMH. In the exploratory analysis, low-probability cycles showed no protocol differences; among high-probability cycles only one association survived correction (long-acting GnRH-agonist fresh transfer vs HRT frozen transfer; absolute difference +48%, 95% CI 28–67%; FDR p=0.002), persisting in the baseline-only analysis.

**Conclusions:**

In women with DOR, an interpretable logistic-regression model provided moderate, internally and temporally validated discrimination for cumulative clinical pregnancy at the pre-transfer decision point, though calibration drifted over time and warrants recalibration before use. The single protocol-related association is hypothesis-generating only. The model is best regarded as a pre-transfer prognostic counselling tool, not a basis for protocol selection, and requires prospective external validation.

**Clinical trial number:**

Not applicable.

**Supplementary Information:**

The online version contains supplementary material available at https://doi.org/10.1186/s12911-026-03709-5.

## Introduction

Ovarian reserve depletion (DOR) represents a significant challenge in reproductive medicine, characterized by a decline in the quantity and quality of oocytes available for fertilization [1, 2]. Patients with DOR typically present with anti-Müllerian hormone (AMH) levels ≤ 1.1 ng/mL [3], and they experience significantly lower success rates in assisted reproductive technology (ART) cycles compared to women with normal ovarian reserve [4, 5].The global prevalence of DOR is increasing, affecting approximately 10% of women seeking fertility treatment, with a marked rise in incidence among women over the age of 35 [6]. The management of DOR patients, particularly those undergoing ART, remains a clinical challenge. These patients often require multiple treatment cycles, experience higher rates of cycle cancellation, poor ovarian response, and lower pregnancy rates [7]. Given these clinical realities, accurate prediction of treatment outcomes in DOR patients holds profound clinical significance across multiple dimensions. First, individualized prognostic information enables realistic expectation management through patient-centered counseling, facilitating informed decision-making between pursuing further treatment versus exploring alternative family-building options. Second, reliable outcome prediction supports optimal resource allocation by identifying patients most likely to benefit from intervention—a critical consideration given the substantial financial burden and psychological toll associated with ART. Third, risk stratification supports individualized prognostic counseling, helping clinicians and patients set realistic expectations and decide on the intensity of counseling rather than selecting an initial ovarian-stimulation protocol [8].

Currently, most IVF/ICSI outcome prediction models rely on single biomarkers such as AMH, follicle count, and female age, which, despite being widely recognized as important prognostic indicators, have limited predictive power, particularly for DOR patients [9, 10]. Cumulative clinical pregnancy rate (cCPR), which reflects the overall success of all embryo transfers from a single egg retrieval, is influenced by a range of variables, including demographic, endocrine, ovarian stimulation, embryological, and male factors [11]. The complex interactions among these variables make traditional predictive models less applicable to DOR patients [12]. As such, there is an urgent need for more sophisticated analytical approaches that can integrate multiple factors to improve the accuracy of treatment outcome predictions for this patient population.

Recent advances in machine learning (ML) offer transformative potential to overcome the inherent limitations of conventional statistical approaches. ML algorithms can autonomously identify complex, non-linear patterns within high-dimensional clinical data and have demonstrated superior predictive performance across diverse medical applications [13, 14]. However, the “black box” nature of sophisticated ML models remains a substantial barrier to clinical implementation, as transparency regarding the underlying basis for predictions is essential for both clinician trust and informed patient decision-making [15, 16].SHapley Additive exPlanations (SHAP), rooted in cooperative game theory, addresses this critical limitation by quantifying individual feature contributions to specific predictions, thereby rendering complex models interpretable without sacrificing predictive accuracy [17].

Beyond prediction alone, the principal aim of this study was to explore heterogeneity in protocol-associated cumulative clinical pregnancy across ovarian stimulation and embryo-transfer strategies in women with DOR. The objectives were: [1] to explore, in a hypothesis-generating manner, whether protocol-associated clinical-pregnancy rates differ across baseline-prognosis risk strata; [2] to derive those risk strata using an interpretable, internally validated machine-learning model; and [3] to identify the most influential predictors through systematic feature selection and SHAP analysis. This study aims to support individualized prognostic counseling and to generate hypotheses for prospective evaluation; it is not intended to establish optimized treatment or evidence-based protocol selection, which would require prospective, randomized trials. Our contribution is therefore applied and practical rather than methodological: we provide a DOR-specific, internally and temporally validated, fully transparent prognostic tool built only from routinely collected variables, together with an explicit, multiplicity-controlled framework for generating—rather than confirming—protocol-related hypotheses. We deliberately show that a parsimonious logistic-regression model matches more complex algorithms, prioritising the transparency, calibration and reproducibility that clinical decision-support in reproductive medicine requires.

## Methods

### Study design and setting

This retrospective cohort study was conducted at the Reproductive Medicine Center of the First Affiliated Hospital of Guangxi Medical University. Clinical and laboratory data were extracted from the institutional electronic database for women undergoing ART between January 2019 and July 2024. Reporting followed the STROBE guideline for observational studies and the TRIPOD guidance for prediction-model studies [18].

**Fig. 1:**
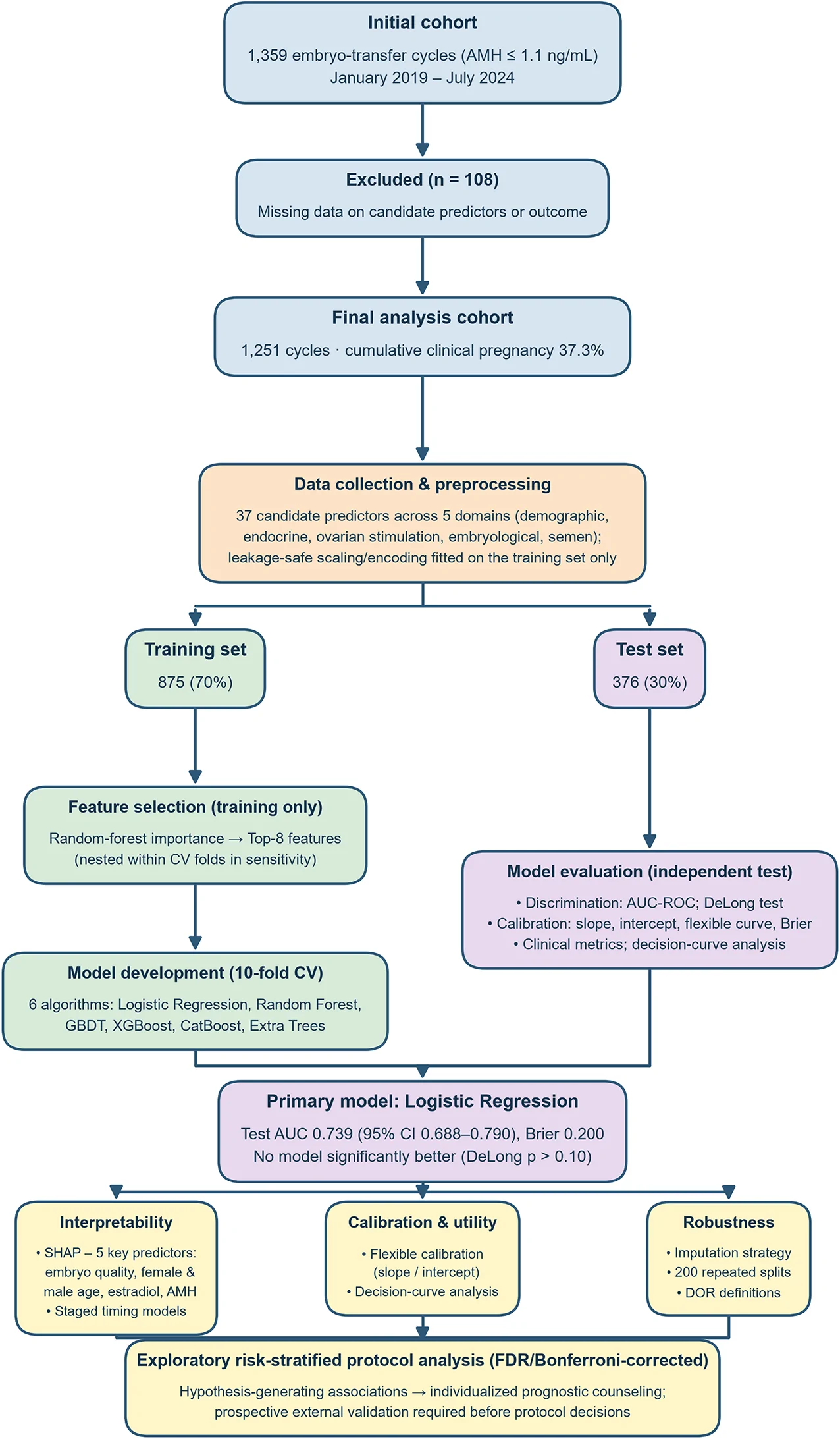
Study design and analytical workflow

The overall study workflow is illustrated in Fig. 1.

### Study population and eligibility criteria

Eligible participants were women with DOR, defined by a serum AMH level of ≤ 1.1 ng/mL, who underwent fresh or frozen-thawed embryo transfer at our center between January 2019 and July 2024; records were retrieved from the institutional electronic medical record system. Cycles were eligible when both the baseline clinical/laboratory data and the pregnancy outcome were documented, and cycles with any missing value among the 37 candidate predictors were not analysed. This dataset is shared with a companion study of cumulative live birth in the same cohort [19]; the present work addresses a different endpoint (clinical pregnancy) and a different question (risk-stratified comparison of stimulation and embryo-transfer protocols), and reaches distinct conclusions. We defined DOR by a serum AMH level ≤ 1.1 ng/mL because AMH was uniformly available across the full study period, is the most reproducible single ovarian-reserve marker in our electronic record, and aligns with the comparator literature, facilitating comparison. We acknowledge that AMH alone does not fully capture the clinically heterogeneous DOR/poor-responder population; baseline antral follicle count and the components required for formal Bologna or POSEIDON classification were not consistently recorded across the study period and could not be applied retrospectively. To mitigate this, we report a DOR-definition sensitivity analysis across AMH ≤ 1.1, a stricter AMH ≤ 0.5, AMH ≤ 1.1 combined with age ≥ 35 (approximating a Bologna/POSEIDON-like criterion), and age ≥ 35 alone (Sensitivity Analyses).

### Primary outcome

The outcome was cumulative clinical pregnancy, assessed per oocyte-retrieval cycle: a retrieval was counted as positive if any resulting transfer (fresh or frozen-thawed) achieved a clinical pregnancy, defined sonographically as an intrauterine gestational sac with fetal cardiac activity. In a conservative variant, retrievals with cryopreserved embryos still untransferred at the end of follow-up were treated as non-pregnant, providing a lower-bound estimate. The analytical unit throughout this study is the oocyte-retrieval cycle; we use the term retrieval cycle consistently and reserve transfer-cycle type (fresh vs. frozen-thawed) as a categorical predictor variable, not as the analytical unit. Cumulative clinical pregnancy was chosen as the primary endpoint because it is ascertained reliably and promptly within the electronic record at our centre, integrates outcomes across all transfers from a single retrieval, and is the most actionable endpoint for the short-horizon, pre-transfer counselling this model is intended to support; long-horizon obstetric follow-up to live birth was less consistently captured across the study period. We emphasise, however, that clinical pregnancy is an intermediate outcome that may overestimate ultimate reproductive success, particularly in older women with DOR who have a higher miscarriage risk; accordingly, the present model must not be interpreted as a live-birth prediction tool. Cumulative live birth in the same cohort is addressed in a separate companion analysis.

### Data collection and variable definition

Thirty-seven baseline candidate predictors were assembled across five clinical domains: patient demographics and reproductive history, endocrine/ovarian-reserve markers, ovarian-stimulation response, embryology, and semen quality.

Demographic and reproductive-history variables comprised female and male age, body-mass index, duration and type of infertility, gravidity, parity, prior abortions, and transfer-cycle type (fresh vs. frozen-thawed).

Endocrine and ovarian-reserve markers were AMH, luteinizing hormone, estradiol, and progesterone.

Ovarian-stimulation response was captured by trigger-day follicle counts (14–16 mm, 16–18 mm, ≥ 18 mm), total follicle count, and the number of oocytes retrieved.

Endometrial assessment comprised endometrial thickness and ultrasonographic pattern (type A/B/C).

Embryology variables were the number of embryos transferred, developmental stage at transfer (cleavage vs. blastocyst), embryo-quality grade, and fertilization method (IVF vs. ICSI).

Semen parameters were recorded before and after processing: pre-wash abstinence, volume, sperm concentration, viability, progressive motility, normal morphology, total count, and total progressively motile count; and post-wash concentration, viability, and progressive motility.

### Data Preprocessing

#### Variable classification and transformation

For the training-versus-test comparison, variables were kept on their native clinical scales; continuous variables were summarized as mean ± SD or median [IQR] according to distribution, and categorical variables as counts and percentages.

For modelling, continuous predictors were standardized to z-scores and categorical predictors integer-encoded; both transformations were fitted on the training set alone and then applied to the test set to prevent information leakage, while original scales and labels were retained for reporting and interpretation.

### Missing data strategy

The primary analysis used complete cases: any cycle with a missing value among the 37 predictors was excluded, which avoided imputation assumptions.

### Train-test split and cross-validation strategy

The cohort was divided 70:30 by outcome-stratified random sampling at the cycle level into a training set and a held-out test set; the test set was reserved solely for final evaluation. Because no patient identifier was available, cycles potentially contributed by the same woman could not be assigned to the same partition; we therefore describe the held-out test set as a cycle-level internal validation set rather than a fully independent cohort. To address this, we additionally performed an out-of-time validation using calendar year (Methods, Sensitivity Analyses).

Within the training set, models were developed and internally validated using outcome-stratified 10-fold cross-validation, so that each observation served once as validation data.

### Feature selection

To mitigate dimensionality and overfitting, predictor importance was evaluated with a random-forest model (mean decrease in Gini impurity), using the training set only. The eight features with the highest importance were retained for modelling. In a sensitivity analysis, feature selection was repeated within each cross-validation fold; mean AUC changed by no more than 0.001, indicating negligible selection-induced optimism.

### Machine learning algorithms

Six classification algorithms representing diverse modeling paradigms were evaluated:

1. **Logistic Regression:** A linear probabilistic model with L2 regularization, maximum iterations=1000, using the liblinear solver [20].
2. **Random Forest**: An ensemble method combining multiple decision trees with bootstrap aggregating and random feature subsampling [21].
3. **Gradient Boosting Decision Trees (GBDT)**: A sequential ensemble technique that builds trees iteratively to correct previous model errors [22].
4. **XGBoost:** An optimized gradient boosting framework with regularization and efficient tree construction, configured with log-loss evaluation metric [23].
5. **CatBoost**: A gradient boosting algorithm with ordered boosting and categorical feature handling, operated in silent mode [24].
6. **Extra Trees**: An ensemble of randomized decision trees using random thresholds for splits, increasing diversity and reducing variance [25]. To avoid optimistic bias from hyperparameter search on a moderate-sized cohort and to maximise reproducibility, we did not perform a hyperparameter grid/random search; instead, all six algorithms were fitted at their documented library default hyperparameters (with only the random seed, and where applicable the solver/evaluation-metric/silent-mode settings noted above, fixed). Model selection among the six was based on cross-validated and held-out discrimination, calibration and clinical utility rather than on tuning. The complete final hyperparameter set for every algorithm (the full scikit-learn/XGBoost/CatBoost parameter dictionaries) is provided in Supplementary Table S3 and in the machine-readable reproducibility file, so that the entire pipeline can be reproduced exactly under the reported software versions and seed (654321).

### Model evaluation

Models were evaluated using 10-fold cross-validation and a held-out (cycle-level internal validation) test set. Discriminative performance was assessed using the area under the receiver operating characteristic curve (AUC-ROC), with 95% confidence intervals calculated via 2,000 bootstrap resamples. To provide a comprehensive assessment, additional metrics included: accuracy (overall classification rate), sensitivity (true positive rate), specificity (true negative rate), positive predictive value (PPV/precision), negative predictive value (NPV), F1-score (harmonic mean of precision and recall), and Matthews correlation coefficient (MCC), a balanced metric that incorporates all elements of the confusion matrix and ranges from − 1 to + 1 [26]. Calibration was assessed using calibration curves and Brier scores [27]. Clinical utility was evaluated through decision curve analysis (DCA) [28].

### Model interpretability

To enhance clinical interpretability, we applied SHapley Additive exPlanations (SHAP) to the primary model. For tree-based algorithms a TreeExplainer was used; for the linear primary model a linear explainer was used. SHAP values quantify each feature’s contribution to individual predictions. Any apparent thresholds (for example near 35 and 40 years) are model-derived and are not clinical cut-offs. The framework included global importance (mean absolute SHAP), beeswarm summary plots, dependence plots, and individualized waterfall/force plots.

### Treatment effect analysis

To explore heterogeneous associations, we performed a risk-stratified comparative analysis. Test-set cycles were dichotomized at the median predicted probability of the primary model (Low vs. High probability). Within each stratum, clinical-pregnancy rates were compared across protocols (Chi-square or Fisher’s exact test; protocols with > = 30 total patients, pairwise comparisons only between protocols with > = 15 patients per stratum). Absolute risk differences with 95% CIs were estimated by bootstrap resampling (1000 iterations). P-values were corrected for multiplicity using the Benjamini-Hochberg false-discovery-rate and Bonferroni methods [29]. Because the strata derive from a model that incorporates post-stimulation and embryo variables (themselves affected by the protocol), this analysis is susceptible to collider/treatment-induced confounding and is reported as exploratory and associative only. To assess vulnerability to treatment-induced (collider) bias, we additionally repeated the within-stratum protocol comparison after re-deriving risk strata from a logistic-regression model that used pre-treatment variables only (excluding all post-stimulation and embryo predictors); per-protocol denominators within each stratum are reported in full.

Out-of-time (temporal) validation. We extracted calendar year from the registration code (REGCOD prefix) and conducted an out-of-time sensitivity analysis: cycles registered in 2019–2022 (*n* = 1,027) formed the temporal training set and cycles registered in 2023–2024 (*n* = 224) the temporal test set. The same leakage-safe pipeline (scaling, encoding and random-forest Top-8 feature selection fit on the temporal training set only) and the same primary logistic-regression model were used. We report temporal test AUC with 2,000-bootstrap 95% CI, calibration slope/intercept, and the Brier score; given the absence of patient identifiers, this remains a cycle-level (not patient-level) external validation.

### Statistical analysis

The characteristics of the training and test sets were compared using appropriate statistical tests. For continuous variables, normality was assessed using the Shapiro-Wilk test (α = 0.05). For normally distributed variables, Levene’s test was used to evaluate variance homogeneity, followed by Student’s t-test for equal variances and Welch’s t-test for unequal variances. Non-normally distributed continuous variables were compared using the Mann-Whitney U test. Categorical variables were analyzed using the Chi-square test of independence for contingency tables, with Fisher’s exact test applied when expected cell frequencies were less than 5 in 2 × 2 tables.

Results are presented as mean +/- SD, median [IQR], or n (%) as appropriate. Analyses used Python 3.11 (scikit-learn 1.9.0, XGBoost 3.2.0, CatBoost 1.2.10, LightGBM 4.6.0, SHAP 0.51.0, statsmodels 0.14.6; random seed 654321) [30, 31]. All tests were two-sided; *P* < 0.05 was significant, with multiple-comparison correction applied to the treatment-effect analysis. Between-model AUCs were compared with the DeLong test [32].

## Results

### Study Population

A total of 1,359 oocyte-retrieval cycles in women with diminished ovarian reserve (AMH ≤ 1.1 ng/mL) were identified between January 2019 and July 2024. After excluding cycles with any missing value among the candidate predictors, 1,251 retrieval cycles were included in the final analysis. These retrieval cycles were divided 70:30 by outcome-stratified random cycle-level sampling into a training set (*n* = 875) and a held-out test set (*n* = 376); the held-out set is a cycle-level internal validation sample rather than a fully independent cohort, because the absence of patient identifiers precluded patient-level partitioning.

Overall, demographic, endocrine, ovarian stimulation, and embryological characteristics were highly comparable between the training and test cohorts. The median female age was 37 years in the training cohort and 38 years in the test cohort (*P* = 0.506), while the median male age was 38 years in both cohorts (*P* = 0.998). Estradiol levels were comparable between cohorts (training: 1079.00 pg/mL vs. test: 1098.00 pg/mL, *P* = 0.955). The median number of oocytes retrieved was 5 in the training cohort and 4 in the test cohort (*P* = 0.975), and the embryo quality distribution was similar between the two cohorts (*P* = 0.983)(Table 1).

**Table 1:** Baseline characteristics of the Top-8 predictors in the training and test cohorts

Predictor	Training (*N* = 875)	Test (*N* = 376)	*P* value
Female age (years)	37.00 [34.00, 41.00]	38.00 [34.00, 40.25]	0.506
Male age (years)	38.00 [34.00, 42.00]	38.00 [34.00, 42.00]	0.998
Embryo quality, n (%)			0.983
Grade 1	126 (14.4%)	56 (14.9%)	
Grade 2	178 (20.3%)	77 (20.5%)	
Grade 3	149 (17.0%)	67 (17.8%)	
Grade 4	280 (32.0%)	114 (30.3%)	
Grade 5	142 (16.2%)	62 (16.5%)	
Estradiol (pg/mL)	1079.00 [668.00, 1712.50]	1098.00 [639.70, 1670.50]	0.955
Luteinizing hormone (IU/L)	2.35 [1.46, 4.04]	2.31 [1.49, 4.08]	0.809
Oocytes retrieved (n)	5.00 [2.00, 7.00]	4.00 [2.00, 7.00]	0.975
Body mass index (kg/m²)	22.50 [20.59, 24.50]	21.80 [20.10, 24.36]	0.017
Anti-Müllerian hormone (ng/mL)	0.64 [0.41, 0.92]	0.65 [0.45, 0.90]	0.717

Continuous variables: mean ± SD (normal) or median [IQR] (non-normal); compared by t/Welch test or Mann-Whitney U. Categorical: n (%); compared by chi-square or Fisher’s exact test

Importantly, cCPR was 37.37% (327 of 875) in the training set and 37.23% (140 of 376) in the test set, with no significant difference between groups (*P* = 1.000), indicating that the training and test cohorts were representative and comparable(Table 1).

### Feature selection and variable importance

**Fig. 2:**
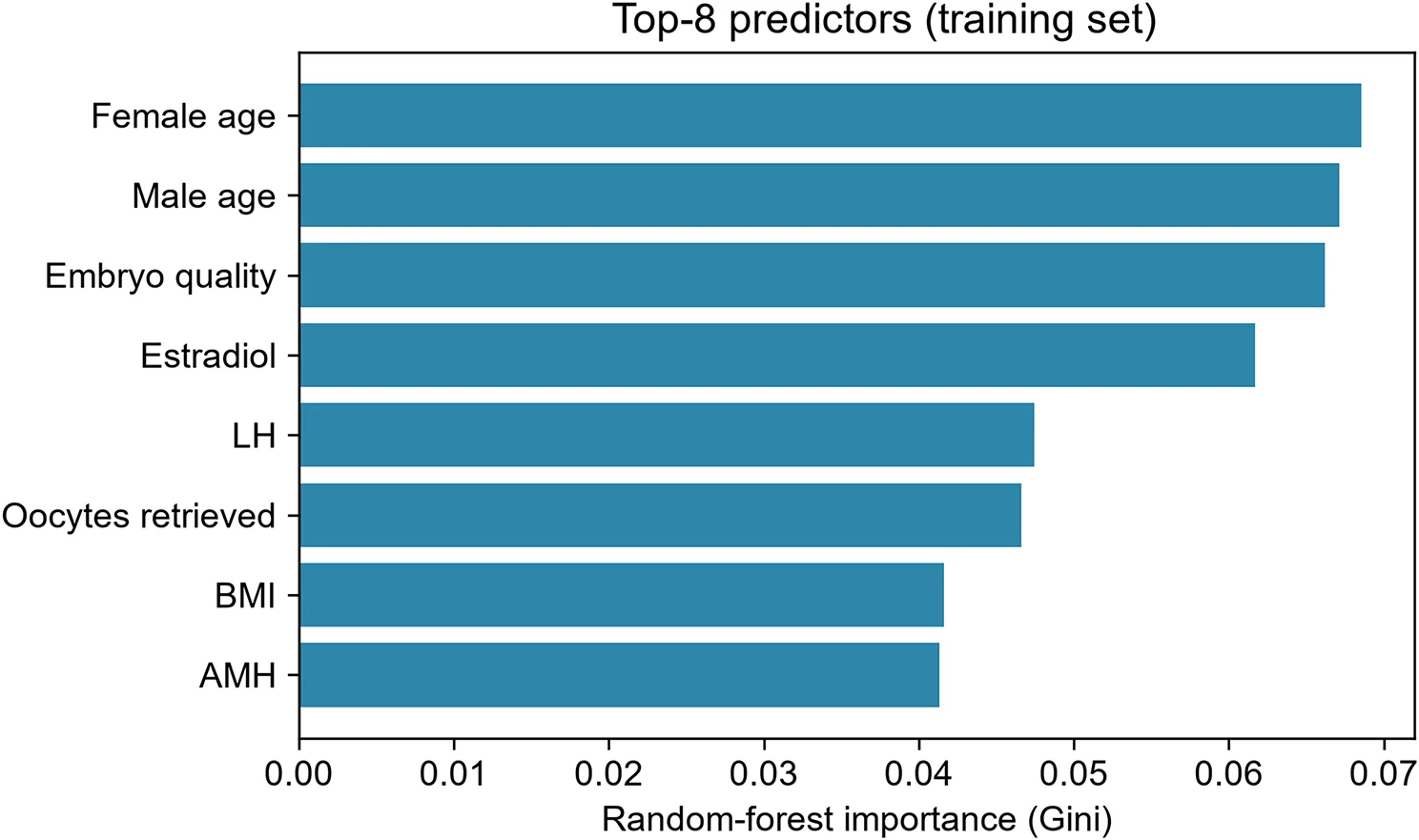
Top-8 predictors ranked by random-forest importance (training set)

To reduce dimensionality, predictor importance was ranked with a random forest trained on the development cohort. The eight most important predictors were retained for modelling (female age, male age, embryo quality, estradiol, luteinizing hormone, oocytes retrieved, body mass index and AMH; Fig. 2). The five most influential by SHAP (embryo quality, female age, male age, estradiol and AMH) are highlighted in the interpretability analysis.

### 10-Fold cross-validation performance on the training set

Six machine learning algorithms were evaluated using 10-fold cross-validation on the training cohort (*N* = 875): Logistic Regression, Random Forest, Gradient Boosting, XGBoost, CatBoost, and Extra Trees (Table 2).

**Table 2:** 10-Fold Cross-Validation Performance Metrics in the training set

Model	Accuracy	AUC	AUC 95% CI	Sensitivity	Specificity	PPV	NPV	F1-Score	MCC
Logistic Regression	0.729	0.803	[0.705, 0.890]	0.560	0.830	0.668	0.761	0.606	0.409
Random Forest	0.711	0.785	[0.683, 0.877]	0.526	0.821	0.643	0.746	0.573	0.367
Gradient Boosting	0.713	0.766	[0.658, 0.862]	0.569	0.799	0.630	0.758	0.596	0.377
XGBoost	0.723	0.770	[0.664, 0.865]	0.591	0.803	0.642	0.768	0.613	0.402
CatBoost	0.719	0.798	[0.698, 0.886]	0.563	0.812	0.651	0.758	0.598	0.391
Extra Trees	0.719	0.792	[0.690, 0.883]	0.535	0.829	0.656	0.750	0.585	0.384

Results are presented as mean across 10 folds. AUC 95% CI calculated using bootstrap method (2000 resamples)

AUC = Area Under the Curve; PPV = Positive Predictive Value; NPV = Negative Predictive Value; MCC = Matthews Correlation Coefficient

On 10-fold cross-validation, Logistic Regression and CatBoost achieved the highest mean AUC (both ~ 0.799), followed by Random Forest (0.786), Extra Trees (0.785), Gradient Boosting (0.783) and XGBoost (0.770); differences among the leading models were small (Table 2).

**Fig. 3:**
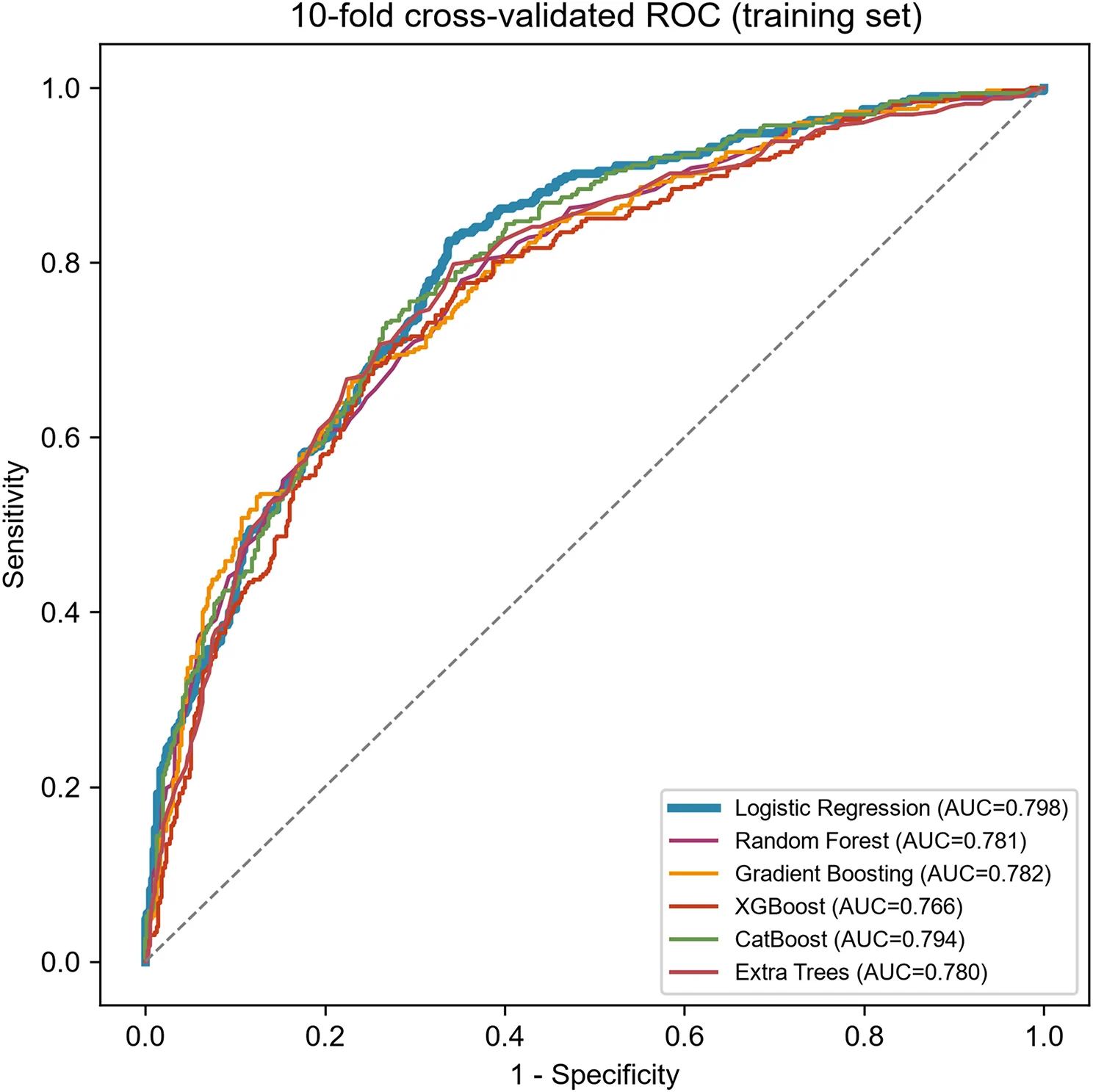
ROC Curve Analysis of Six Machine-Learning Models in 10-Fold Cross-Validation

Cross-validation ROC curves were consistent across folds (Fig. 3), with overlapping confidence intervals indicating no clear superiority of any single algorithm.

### Performance evaluation on the test set

All six algorithms were then evaluated on the held-out test set (*N* = 376), which was not used for training, feature selection, preprocessing fit, or hyperparameter tuning (Table 3). Performance on this set is reported as cycle-level internal validation; out-of-time and baseline-only sensitivity analyses are reported below.

**Table 3:** Performance Evaluation on the test set

Model	Accuracy	AUC	95% CI (AUC)	Sensitivity	Specificity	PPV	NPV	F1-Score	MCC
Logistic Regression	0.707	0.742	[0.692, 0.792]	0.486	0.839	0.642	0.733	0.553	0.349
Random Forest	0.710	0.729	[0.677, 0.778]	0.500	0.835	0.642	0.738	0.562	0.357
Gradient Boosting	0.681	0.719	[0.666, 0.769]	0.507	0.784	0.582	0.728	0.542	0.301
XGBoost	0.662	0.718	[0.664, 0.770]	0.507	0.754	0.550	0.721	0.528	0.266
CatBoost	0.710	0.743	[0.692, 0.793]	0.521	0.822	0.635	0.743	0.573	0.360
Extra Trees	0.702	0.742	[0.689, 0.790]	0.486	0.831	0.630	0.731	0.548	0.338

Final evaluation on independent test set (30% of data). AUC 95% CI calculated using bootstrap method (2000 resamples)

**Fig. 4:**
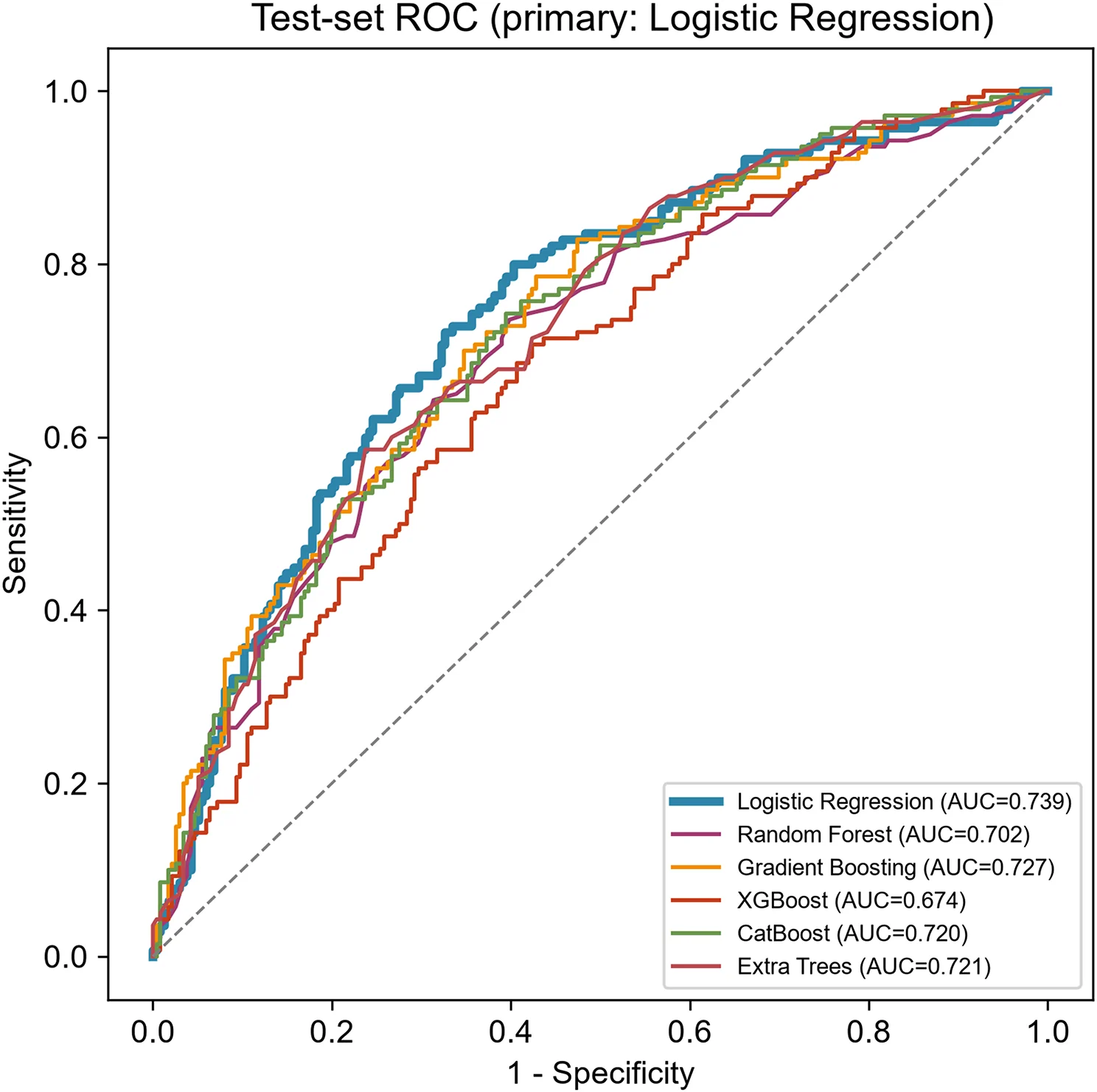
Test-set ROC curves for six models; logistic regression (primary)

On the held-out test set (cycle-level internal validation), Logistic Regression showed the highest discrimination (AUC = 0.739, 95% CI 0.688–0.790; the primary model, a logistic regression fitted on the eight selected features), followed by Gradient Boosting (0.727), Extra Trees (0.724), Random Forest (0.721) and CatBoost (0.720); XGBoost was lowest (0.674). Pairwise DeLong tests [32] showed no significant difference between Logistic Regression and CatBoost (*p* = 0.11), Gradient Boosting (*p* = 0.38), Extra Trees (*p* = 0.28) or Random Forest (*p* = 0.19), whereas Logistic Regression exceeded XGBoost (*p* = 0.001) (Fig. 4; Table 3).

For the primary model (Logistic Regression), accuracy was 0.697, sensitivity 0.493, specificity 0.818, F1 0.548 and MCC 0.328 (Table 3). Given comparable discrimination across the leading algorithms, the parsimonious and fully interpretable Logistic Regression was adopted as the primary model. These metrics indicate moderate discrimination with limited sensitivity at the 0.5 threshold; we therefore frame the model as supporting probabilistic counselling rather than binary clinical decisions.

From cross-validation to the held-out test set the AUC decreased modestly (Logistic Regression, ~ 0.799 to 0.739), consistent with the expected optimism of internal model development rather than material overfitting.

### Model calibration and clinical utility assessment

#### Calibration performance

**Fig. 5:**
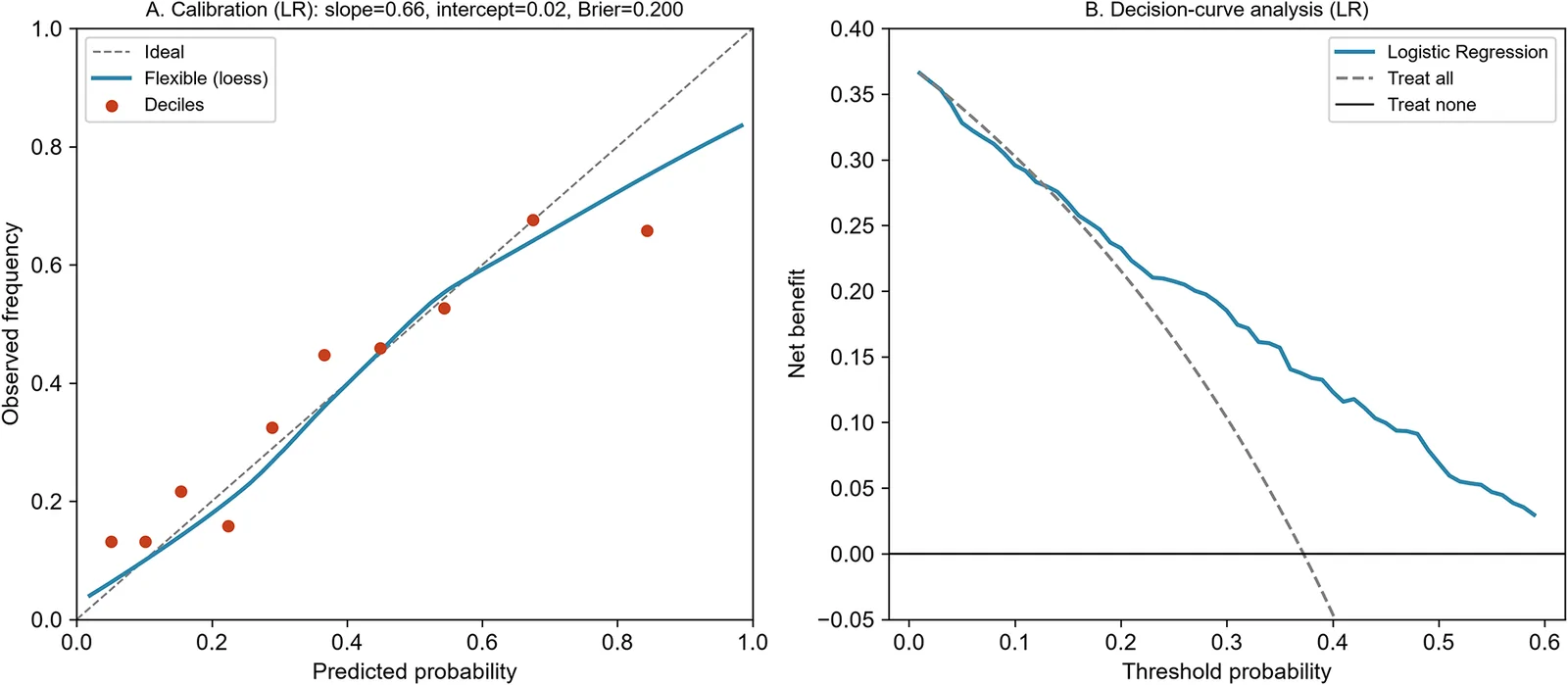
(**A**) Flexible calibration of the primary model (Logistic Regression); (**B**) decision-curve analysis

Calibration was assessed using the calibration slope, calibration-in-the-large (intercept), a flexible (loess) calibration curve and the Brier score [33] (Fig. 5A). For the primary model the Brier score was 0.200, the calibration-in-the-large intercept 0.02 (near-ideal), and the calibration slope 0.66, indicating mild over-dispersion of predicted risks. Among all models, Logistic Regression had the best Brier score and calibration slope, whereas XGBoost was poorly calibrated (slope 0.20, intercept 0.44). We did not rely on a non-significant Hosmer-Lemeshow test alone to claim adequate calibration. Because the calibration slope (0.66) indicates mild over-dispersion of predicted risks, we recommend recalibration (e.g., logistic recalibration or shrinkage of coefficients) before any prospective application; we report the slope/intercept transparently rather than rely on global tests alone.

### Decision curve analysis

Decision-curve analysis (Fig. 5B) evaluated the net benefit of using the model to decide whether to proceed with, or intensively counsel before, transfer in a given cycle. Across the clinically relevant threshold range (~ 0.15–0.40) the models provided positive net benefit, exceeding both treat-all and treat-none strategies; net benefit declined above 0.5, consistent with the 37% event rate. Operationally, the threshold range maps to two counselling actions rather than a treatment-selection rule: at predicted probabilities below the lower threshold, we suggest more intensive counselling regarding realistic expectations, the option of cumulative cycles, and alternative family-building options; within the range we suggest standard counselling and proceeding to transfer; we do not propose using the model to choose between stimulation protocols.

Considering discrimination, calibration and clinical utility together, the parsimonious Logistic Regression provided performance comparable to the more complex algorithms and was adopted as the primary model.

### SHAP interpretability analysis

**Fig. 6:**
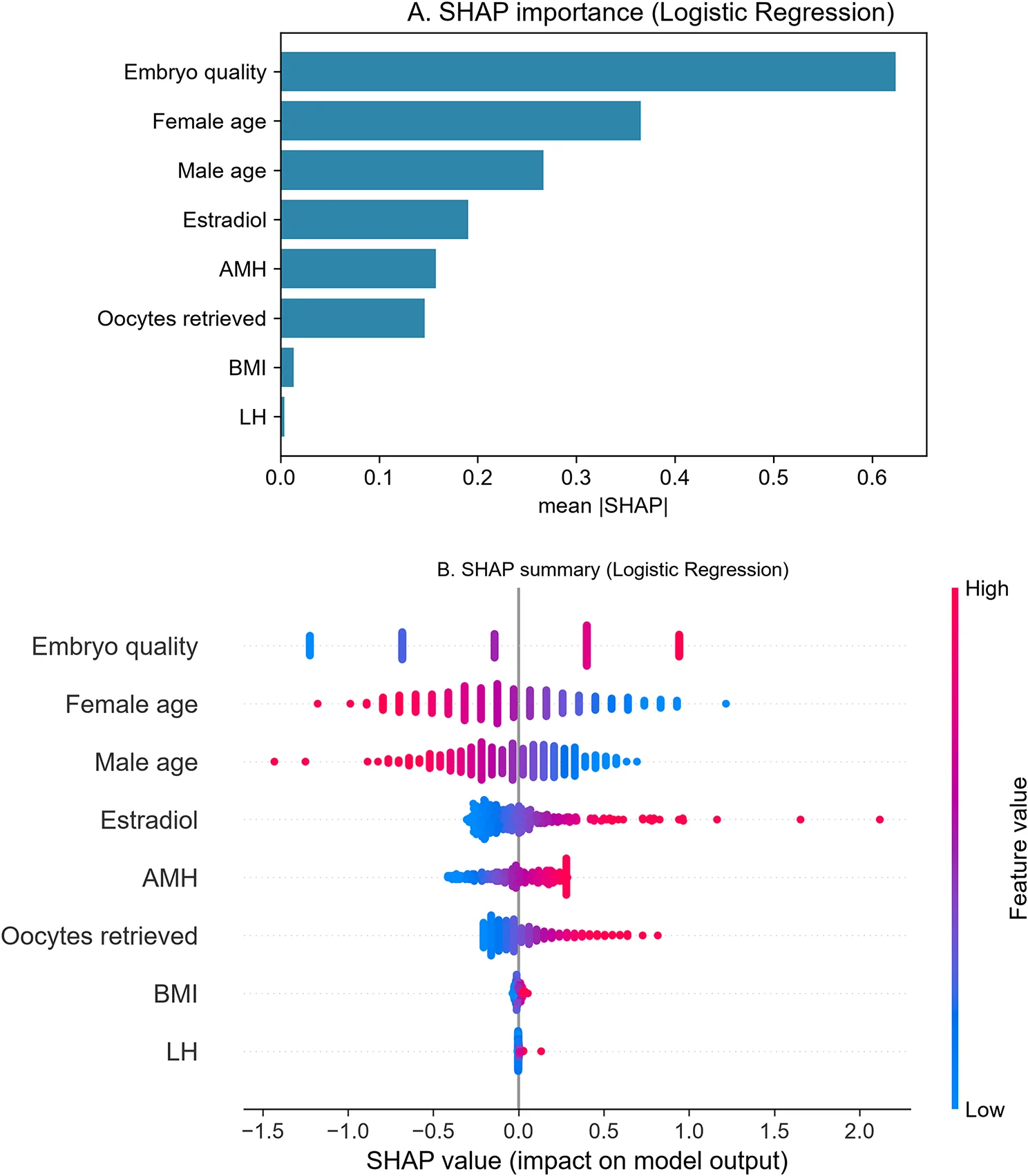
(**A**) SHAP importance and (**B**) SHAP summary for the primary Logistic-Regression model

SHAP analysis of the primary (Logistic Regression) model (Fig. 6A) identified embryo quality as the most influential predictor (mean |SHAP|=0.62; higher quality associated with higher predicted pregnancy), followed by female age (0.37; negative), male age (0.27; negative), estradiol (0.19; positive) and AMH (0.16; positive); oocytes retrieved ranked closely thereafter. SHAP quantifies each feature’s contribution to the model’s predictions; it does not establish biological mechanisms or causal effects, and the rankings should be interpreted as descriptive of the fitted model, not as confirmation of clinical causation.

In the linear primary model these associations were monotonic. In an exploratory analysis, tree-based models (e.g., CatBoost) suggested possible non-linear inflections - an accentuated decline in predicted pregnancy beyond approximately 35 years (female) and 40 years (male), and a plateau in the effect of estradiol and oocyte number (Fig. 6B). These did not improve discrimination over the linear model (DeLong *p* > 0.10) and are reported only as model-derived, hypothesis-generating observations.

Individual prediction explanations via SHAP waterfall plots illustrated model decision-making for three cases.

### Sensitivity analyses

Sensitivity analyses confirmed robustness. Imputation strategy (mean/median/complete-case) had negligible impact, and performing feature selection within each cross-validation fold changed AUC by < = 0.001. Across 200 repeated random train-test splits, test AUC was stable (mean 0.78, 2.5–97.5% 0.74–0.82), with the primary split at the conservative end. Performance was also consistent across alternative DOR definitions (AMH ≤ 1.1, AMH ≤ 0.5, AMH ≤ 1.1 with age ≥ 35, and age ≥ 35; test AUC 0.735–0.753).

**Fig. 7:**
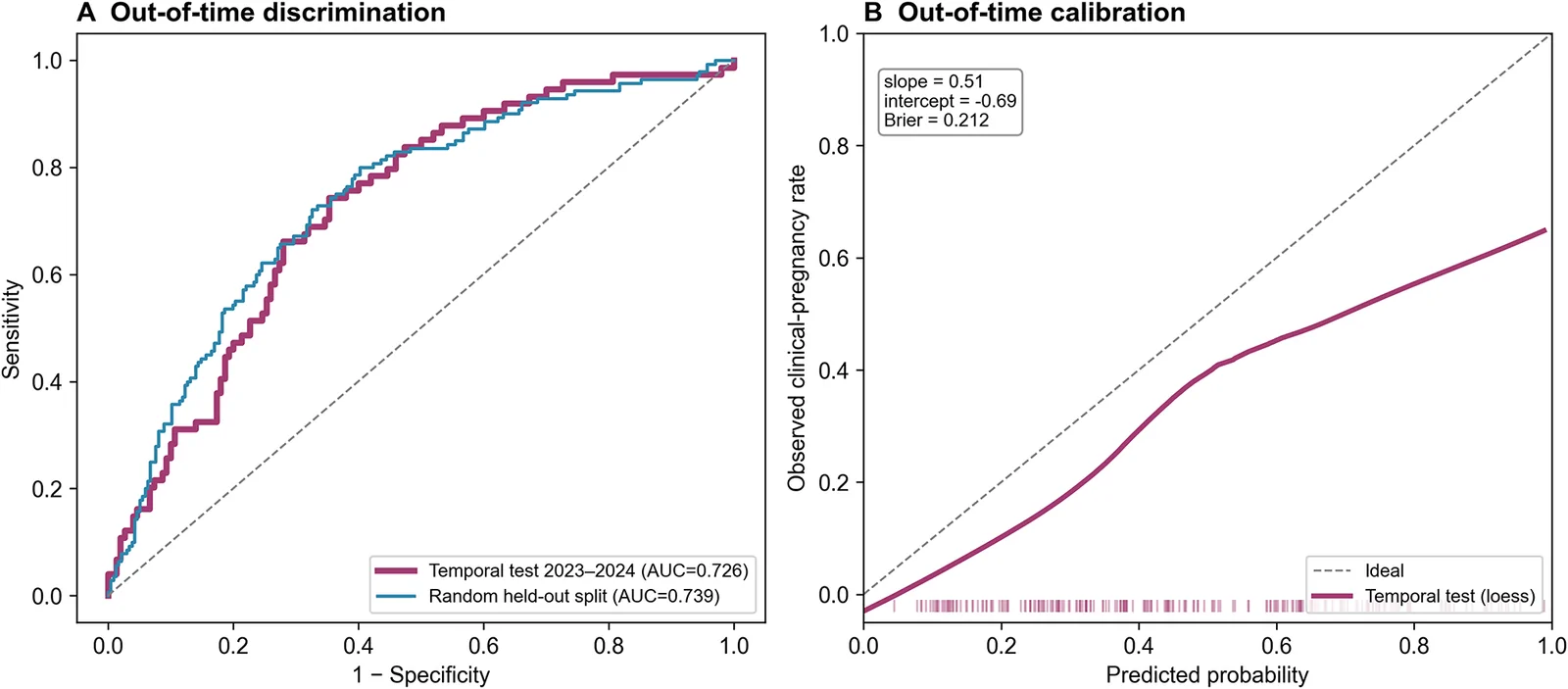
Out-of-time (temporal) validation of the primary logistic-regression model. (**A**) Receiver-operating-characteristic curves on the temporal test set (cycles registered in 2023–2024, *n* = 224; AUC 0.726) compared with the random held-out split (AUC 0.739). (**B**) Flexible (loess) calibration curve on the temporal test set, showing calibration drift over calendar time (slope 0.508; calibration-in-the-large intercept − 0.69; Brier 0.212). Training cycles were registered in 2019–2022 (*n* = 1,027). Because no patient identifier was available, this is an out-of-time, cycle-level validation rather than a patient-level external validation

Because several predictors (estradiol, oocytes retrieved, embryo quality) become available only after stimulation or at transfer, a model restricted to pre-treatment variables (22 predictors) discriminated less well (test AUC 0.683) than a model using all 37 pre-transfer predictors (test AUC 0.756); both of these staged models differ from the parsimonious eight-feature model adopted throughout as the primary model (test AUC 0.739 on the 70:30 cycle-level split), and index the information available at successive decision points rather than serving as the primary model. The model is therefore intended for use at the pre-transfer decision point. A detailed timing-specific (stage-by-stage) modelling framework is beyond the scope of this report. Baseline-only risk-stratified protocol comparison (sensitivity). To assess vulnerability of the protocol comparison to treatment-induced (collider) bias, we re-derived risk strata from a logistic-regression model fitted on pre-treatment variables only (22 predictors; no estradiol, oocytes retrieved, embryo quality or other post-stimulation/embryo variables). The baseline-only model showed lower discrimination (test AUC 0.683) but partitioned the held-out set evenly (median threshold 0.360; low *n* = 188, high *n* = 188). The headline association observed in the main analysis persisted: in the favourable-prognosis stratum, long-acting GnRH-agonist fresh transfer 75.0% (30/40) versus HRT frozen transfer 23.3% (7/30), absolute difference + 51.7% (95% CI 30.8–70.0); FDR-adjusted *p* = 0.002, Bonferroni-adjusted *p* = 0.002. All other pairwise comparisons were non-significant after correction (raw-significant 4 → FDR 1 → Bonferroni 1), and no comparison was significant in the low-probability stratum. Exact per-protocol denominators within each stratum (range 20–61) are provided in Supplementary Table S2. The persistence of the signal under a stratification scheme that does not condition on post-treatment mediators reduces, but does not eliminate, concern about collider/treatment-induced confounding; non-randomized confounding by indication remains. Out-of-time (temporal) validation. Using calendar year derived from the registration code, we trained on 2019–2022 cycles (*n* = 1,027; clinical-pregnancy rate 38.3%) and tested on 2023–2024 cycles (*n* = 224; rate 33.0%). The primary logistic-regression model preserved discrimination on the temporal test set (AUC 0.726, 95% CI 0.655–0.796), comparable to the random held-out estimate (0.739, 95% CI 0.688–0.790; Fig. 7A). Calibration, however, drifted across time (slope 0.508; calibration-in-the-large intercept − 0.69; Brier 0.212; Fig. 7B), consistent with a lower clinical-pregnancy rate in 2023–2024 and supporting the need for recalibration before any prospective use. Because no patient identifier was available, this is an out-of-time, cycle-level validation rather than a patient-level external validation.

#### Treatment effect analysis

**Fig. 8:**
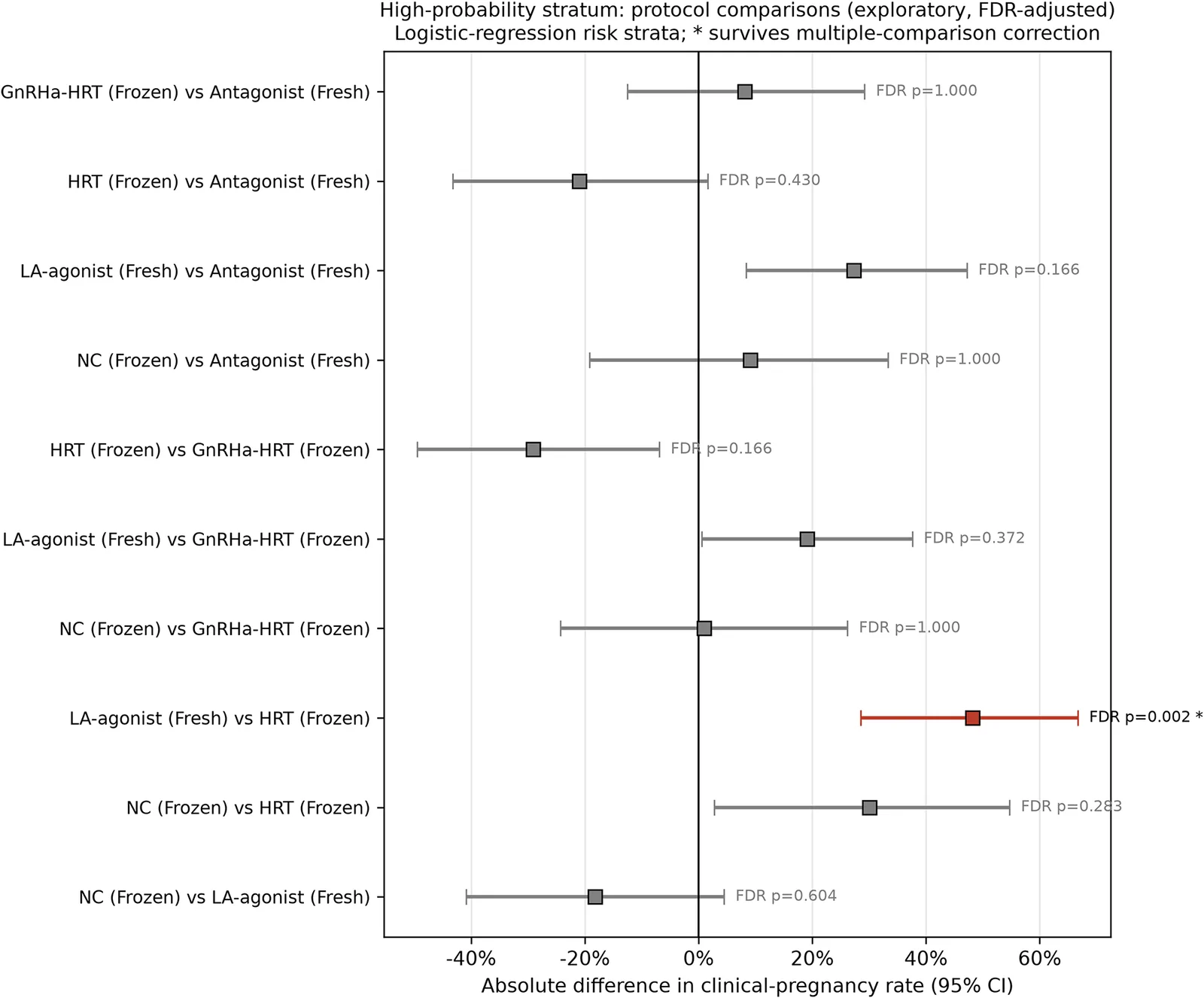
Forest plot of exploratory protocol comparisons in the high-probability stratum (absolute risk differences, 95% CI; FDR-adjusted)

In an exploratory risk-stratified analysis (Fig. 8), patients were dichotomized at the median predicted probability. Among low-probability cycles, no significant differences were observed across treatment protocols.

Among high-probability cycles, the only difference that remained significant after correction for multiple comparisons was a higher clinical-pregnancy rate with a long-acting GnRH-agonist fresh-transfer protocol (77%) than with HRT frozen transfer (29%) (absolute difference + 48%, 95% CI 28–67%; FDR-adjusted *p* = 0.002; Bonferroni *p* = 0.002). Apparent differences versus GnRH-antagonist fresh (+ 27%) and GnRHa-HRT frozen (+ 19%) were not significant after correction (FDR *p* = 0.17). Exact per-protocol denominators within each stratum are reported in Supplementary Table S1. In the high-probability stratum the cell sizes were: long-acting GnRH-agonist fresh transfer 34/44 (77.3%), GnRHa-HRT frozen 25/43 (58.1%), GnRH-antagonist fresh 19/38 (50.0%), NC frozen 13/22 (59.1%) and HRT frozen 9/31 (29.0%); in the low-probability stratum: HRT frozen 11/60 (18.3%), GnRH-antagonist fresh 6/35 (17.1%), GnRHa-HRT frozen 5/34 (14.7%), NC frozen 7/31 (22.6%) and long-acting GnRH-agonist fresh 3/17 (17.6%). Because these subgroups are small, the large absolute differences should be interpreted with caution and regarded as hypothesis-generating rather than as reliable effect estimates.

These associations are exploratory and hypothesis-generating. Because the strata are defined using a model that incorporates post-stimulation and embryo variables, and because treatment was not randomized, the comparisons are susceptible to confounding by indication and collider bias and cannot support causal or treatment-selection conclusions; they indicate signals that warrant prospective, randomized evaluation.

## Discussion

### Principal findings

This study developed and internally validated machine-learning models for cumulative clinical pregnancy in women with DOR. Discrimination was moderate; a parsimonious Logistic Regression (test AUC 0.739, Brier 0.200) performed at least as well as more complex algorithms, which conferred no significant advantage (DeLong *p* > 0.10). The five most influential predictors by SHAP were embryo quality, female age, male age, estradiol and AMH. An exploratory risk-stratified analysis suggested, after multiple-comparison correction, a single protocol-related association among high-probability cycles (long-acting GnRH-agonist fresh transfer vs. HRT frozen transfer), which is hypothesis-generating only. These findings support individualized prognostic counseling but not, on the present evidence, protocol selection. We emphasise that the model uses variables available only by the pre-transfer decision point (notably embryo quality and post-stimulation hormones), and is therefore framed as a pre-transfer prognostic counselling tool: it cannot be used to choose between initial ovarian-stimulation protocols, for which a separate, baseline-only counselling model would be required.

### Comparison with existing literature

#### Prediction model performance

Our primary model’s discrimination (test AUC 0.739) is comparable to recently published IVF prediction models; an analysis of live-birth prediction models reported AUCs of 0.50–0.68 [34], although that endpoint is harder to predict than clinical pregnancy, so this comparison is indicative of general performance ranges rather than a claim of superiority. Most existing models were developed in general IVF populations rather than DOR specifically [3]. Our work is consistent with a broader move toward objective, data-driven and non-invasive assessment in reproductive medicine, including quantitative imaging such as endometrial shear-wave elastography [35]. We focused on a DOR-specific cohort, used cumulative clinical pregnancy at the retrieval-cycle level, and applied leakage-safe feature selection; nevertheless discrimination remained moderate, reflecting the intrinsic difficulty of prediction in this population. Given the moderate discrimination (test AUC 0.739) and limited sensitivity at the 0.5 threshold, the realistic clinical role of the model is to inform probabilistic counselling about the cumulative chance of clinical pregnancy from a given retrieval, to support decisions about the intensity of counselling and about whether to discuss alternative reproductive options at low predicted probabilities; it is not adequate to support pre-treatment protocol selection or binary go/no-go decisions.

Notably, a parsimonious model performed comparably to more complex algorithms, and adding features beyond the leading predictors yielded little gain. This aligns with the principle that simpler models with few high-quality predictors often generalize as well as more complex ones, and that flexible algorithms did not significantly improve discrimination here (DeLong *p* > 0.10). Practically, the model requires only routinely collected clinical data. A practically important reading of these results is that, in this DOR cohort, complex machine-learning algorithms were not necessary: a parsimonious logistic regression matched their discrimination, was better calibrated, and offers the transparency and reproducibility expected of a clinical prediction model. We therefore present parsimony — rather than algorithmic sophistication — as a useful and clinically actionable finding.

### Feature importance and biological plausibility

The SHAP-derived feature importance hierarchy—with embryo quality ranking highest, followed by female age—exhibits strong biological plausibility and consistency with established reproductive physiology. We emphasise that SHAP describes how the fitted model uses each feature; it does not, by itself, demonstrate biological mechanism or causal effect, and the importance ordering should not be over-interpreted as a hierarchy of pathophysiological drivers.

The identification of embryo quality as the most influential predictor strongly aligns with its well-established critical role in determining implantation success and pregnancy outcomes. High-quality embryos exhibit superior developmental competence, implantation potential, and chromosomal integrity compared to lower-grade embryos [36, 37]. The dominant contribution of embryo quality in our DOR cohort underscores that, despite reduced ovarian reserve, embryo developmental competence remains the primary determinant of treatment success. This finding emphasizes the importance of optimizing embryo selection strategies and culture conditions to maximize pregnancy rates in this challenging patient population.

The prominence of female age, and the contribution of male age, accord with evidence that advancing parental age is associated with poorer reproductive outcomes [38–41]. Apparent age inflections near 35 and 40 years emerged only in exploratory tree-based models and are model-derived; they are not validated clinical cut-points and did not improve discrimination over the linear model.

Estradiol (E2) demonstrated moderate predictive value in our model. As a key marker of follicular development and ovarian response, E2 levels reflect the functional capacity of developing follicles and correlate with endometrial receptivity. The intermediate contribution of E2 suggests that while it provides valuable information about ovarian response during stimulation, its predictive value may be partially captured by other correlated markers such as oocyte yield and AMH in multivariable models.

### Treatment effect heterogeneity

An exploratory observation was heterogeneity in protocol-associated pregnancy rates by baseline predicted probability. Among high-probability cycles, a long-acting GnRH-agonist fresh-transfer protocol was associated with a higher pregnancy rate than HRT frozen transfer; this was the only comparison to survive multiple-comparison correction. In a sensitivity analysis using risk strata defined from pre-treatment variables only (Sensitivity Analyses), the same direction and magnitude of association were observed (long-acting GnRH-agonist fresh transfer 75.0% vs. HRT frozen transfer 23.3%; FDR-adjusted *p* = 0.002), reducing — but not eliminating — concern that the signal is purely an artefact of conditioning on post-treatment mediators. Because the original treatment was not randomized, residual confounding by indication remains, and the language throughout is associative.

These associations should not be interpreted causally. The absence of differences among low-probability cycles may reflect intrinsic biological constraints, whereas apparent differences among high-probability cycles may equally reflect confounding by indication, since protocol choice is not random and the strata depend on post-treatment variables.

Several mechanisms could plausibly contribute to such an association (e.g., pituitary suppression reducing premature luteinization, avoidance of freeze-thaw effects, and endometrial-embryo synchrony under physiologic hormones); however, these remain speculative given the observational, exploratory nature of the analysis.

However, we acknowledge that our treatment effect analysis is observational and therefore susceptible to residual confounding by unmeasured patient characteristics or physician selection preferences. The absence of randomization precludes definitive causal inference; although comparisons occur within homogeneous prognostic subgroups, this design reduces but does not eliminate confounding by indication, because the factors driving protocol choice are unmeasured and operate within strata as well. Confirmation through prospective randomized trials stratified by model-predicted risk would provide definitive evidence. Critically, these unmeasured factors are not merely general study limitations but direct threats to the protocol comparison itself: prior ART history, the number of previously failed cycles, infertility diagnosis, the indication for embryo freezing, endometrial-preparation details, clinician preference, and laboratory factors may each influence both the choice of stimulation/transfer protocol and the probability of clinical pregnancy, and therefore act as classic confounders by indication that could fully account for the observed between-protocol differences. Because none of these variables was available, we cannot adjust for them, and the protocol associations must be read as confounded and hypothesis-generating.

### Clinical implications and applications

Our findings have several potential applications. First, the internally validated logistic-regression model could, after external validation, generate individualized pregnancy-probability estimates to support data-driven counseling about realistic expectations and shared decision-making. In terms of contribution to clinical decision-making, this study offers three concrete deliverables rather than a new algorithm: (i) a transparent, well-calibrated pre-transfer counselling model usable from routine data; (ii) evidence that algorithmic complexity is unnecessary in this setting, so a simple logistic regression suffices; and (iii) a reproducible, bias-aware template for exploring protocol-related heterogeneity without overstating causality.

Second, the SHAP-based feature importance analysis identifies modifiable (estradiol levels, oocytes retrieved, potentially modifiable in subsequent cycles) versus non-modifiable (age) risk factors, enabling targeted counseling regarding intervention opportunities. For instance, lower estradiol response or oocyte yield is associated with lower predicted probability and may prompt discussion of subsequent-cycle options, whereas advanced age is associated with poorer prognosis and may warrant counseling about timelines and realistic expectations; these are associative observations, not treatment recommendations.

Third, the risk-stratified analysis is exploratory and does not provide evidence-based guidance for protocol selection. The single association that survived correction should be regarded as a hypothesis for prospective testing and cannot, on current evidence, justify changing protocol choice.

The present analysis and the companion cumulative-live-birth study [19] derive from a largely overlapping single-centre DOR cohort; the small difference in analysable cycles (*n* = 1,251 here versus *n* = 1,234 there) reflects endpoint-specific complete-case filtering rather than a distinct patient population. The two studies differ in endpoint (clinical pregnancy versus live birth), primary question (risk-stratified protocol comparison versus stage-specific prediction) and primary model (logistic regression versus CatBoost); a full dimension-by-dimension comparison is provided in Supplementary Table S4.

### Strengths and limitations

Several limitations warrant consideration. First, this single-center retrospective study limits generalizability, and complete-case selection (108/1359 cycles, 7.9%, excluded for missing data) assumes data were missing completely at random and may introduce selection bias if missingness was related to cycle prognosis (for example, if lower-prognosis cycles had less complete semen or hormone data); external validation is an essential prerequisite to any clinical use, not an optional step. Second, the unit of analysis was the oocyte-retrieval cycle, and the dataset contained no patient identifier, so cycles potentially contributed by the same woman could not be linked and a patient-level (clustered) split was not possible; repeated random splits indicated stable discrimination, but residual non-independence cannot be excluded; because some women may contribute more than one cycle, the effective sample size is likely smaller than *n* = 1,251, and standard errors and confidence intervals may be narrower than they would be under full independence. Third, the primary endpoint was clinical pregnancy, which may overestimate ultimate success relative to live birth (analysed separately), particularly in DOR/older women with higher miscarriage risk. Fourth, DOR was defined by AMH alone, without antral follicle count or Bologna/POSEIDON criteria, although results were stable across alternative definitions. Fifth, important confounders (lifestyle, prior ART history and number of failed cycles, infertility diagnosis, endometrial-preparation details, freezing indication, and clinician/laboratory factors) were unavailable, and the treatment-effect analysis is observational, exploratory, and susceptible to confounding by indication and collider bias. Sixth, although the model preserved discrimination on out-of-time validation (2019–2022 train, 2023–2024 test; AUC 0.726), calibration drifted across calendar time (slope 0.508; intercept − 0.69), so any prospective use will require recalibration. A summary table comparing the present analysis with the companion live-birth study is provided (Supplementary Table S4) to clarify the incremental contribution of this submission.

### Future research directions

Several research directions would extend and validate our findings. First, external validation in independent cohorts from diverse geographic regions, ethnic populations, and clinical practice settings is essential to establish model transportability and identify any population-specific recalibration needs. Multi-center collaborative studies would provide the large-scale data necessary for robust external validation.

Second, prospective implementation studies evaluating clinical impact are needed to determine whether model-guided counseling improves patient outcomes, satisfaction, and resource utilization compared to usual care. Such studies should employ rigorous designs such as cluster randomized trials to minimize contamination bias.

Third, incorporation of advanced biomarkers—including anti-Müllerian hormone trajectory patterns, dynamic endocrine profiling, time-lapse embryo imaging parameters, and molecular markers of endometrial receptivity—may enhance predictive accuracy beyond the routinely collected variables in our current model. Integration of multi-omics data (genomics, transcriptomics, proteomics) represents a longer-term frontier.

Fourth, extension to predict additional outcomes beyond clinical pregnancy—including live birth, pregnancy complications, and multiple gestation—would provide comprehensive risk assessment across the full spectrum of reproductive outcomes. Multi-outcome prediction models that simultaneously estimate probabilities of various endpoints would support holistic counseling.

## Conclusions

In conclusion, an interpretable, parsimonious logistic-regression model provided moderate, internally and temporally validated discrimination (test AUC 0.739; out-of-time AUC 0.726) for cumulative clinical pregnancy in women with DOR, and performed as well as more complex algorithms. Calibration was modest (slope 0.66) and drifted over time, so recalibration would be needed before any clinical use. SHAP identified embryo quality, female age, male age, estradiol and AMH as the most influential predictors. An exploratory risk-stratified analysis generated a single protocol-related hypothesis among high-probability cycles. These hypothesis-generating findings can support individualized pre-transfer prognostic counselling but require prospective external validation before informing protocol selection. 

## Supplementary Information

Below is the link to the electronic supplementary material.

Supplementary Material 1: Supplementary Table S1. Exact per-protocol denominators within each risk stratum — primary risk-stratified analysis (held-out test set; strata defined by the full pre-transfer logistic-regression model). Supplementary Table S2. Exact per-protocol denominators within each risk stratum — baseline-only sensitivity analysis (strata defined by a logistic-regression model using pre-treatment variables only). Supplementary Table S3. Final hyperparameters for each algorithm (library defaults; no hyperparameter search was performed). Supplementary Table S4. Comparison of the present manuscript with the companion live-birth study (Liu et al., Front Endocrinol 2026;17:1832301), based on an overlapping single-centre DOR cohort: the two studies differ in primary endpoint (clinical pregnancy vs live birth), research question (risk-stratified protocol comparison vs stage-specific prediction), primary model (logistic regression vs CatBoost), and conclusions

## Data Availability

The raw clinical ART data are protected and are not available to share due to the data privacy laws of the first affiliated hospital of Guangxi medical university.

## Abbreviations

ART: Assisted reproductive technology
DOR: Diminished ovarian reserve
AMH: Anti-Müllerian hormone
IVF: In vitro fertilization
ICSI: Intracytoplasmic sperm injection
cCPR: Cumulative clinical pregnancy rate
ML: Machine learning
SHAP: SHapley Additive exPlanations
RF: Random forest
GBDT: Gradient boosting decision trees
LR: Logistic regression
AUC: Area under the receiver operating characteristic curve
ROC: Receiver operating characteristic
CI: Confidence interval
MCC: Matthews correlation coefficient
PPV: Positive predictive value
NPV: Negative predictive value
DCA: Decision-curve analysis
FDR: False-discovery rate
GnRH: Gonadotropin-releasing hormone
GnRHa: GnRH agonist
HRT: Hormone replacement therapy
NC: Natural cycle
MS: Mild stimulation
E2: Estradiol
LH: Luteinizing hormone
BMI: Body-mass index
STROBE: Strengthening the Reporting of Observational Studies in Epidemiology
TRIPOD: Transparent Reporting of a multivariable prediction model for Individual Prognosis Or Diagnosis
