# Supplementary material for: Interpretable, internally validated prognostic modelling of cumulative clinical pregnancy in women with diminished ovarian reserve
Source: BMC Med Inform Decis Mak. 2026 Jul 18;26:408. doi: 10.1186/s12911-026-03709-5 (PMC13662997; doi:10.1186/s12911-026-03709-5)
Supplement: Supplementary file 1 [file 12911_2026_3709_MOESM1_ESM.docx]

# Supplementary Tables

**Supplementary Table S1. Exact per-protocol denominators within each risk stratum — primary risk-stratified analysis (held-out test set; strata defined by the full pre-transfer logistic-regression model).**

| **Risk stratum** | **Protocol** | **Clinical pregnancies / cycles (n)** | **Rate (%)** |
| --- | --- | --- | --- |
| High probability | Long-acting GnRH agonist (Fresh) | 34/44 | 77.3 |
| High probability | NC (Frozen) | 13/22 | 59.1 |
| High probability | GnRHa-HRT (Frozen) | 25/43 | 58.1 |
| High probability | GnRH antagonist (Fresh) | 19/38 | 50.0 |
| High probability | HRT (Frozen) | 9/31 | 29.0 |
| Low probability | NC (Frozen) | 7/31 | 22.6 |
| Low probability | HRT (Frozen) | 11/60 | 18.3 |
| Low probability | Long-acting GnRH agonist (Fresh) | 3/17 | 17.6 |
| Low probability | GnRH antagonist (Fresh) | 6/35 | 17.1 |
| Low probability | GnRHa-HRT (Frozen) | 5/34 | 14.7 |

*Strata were defined by the median predicted probability of the primary (full pre-transfer) logistic-regression model. Protocols with <30 total cycles, and within-stratum cells with <15 cycles, were not eligible for pairwise testing. Absolute differences from small cells should be interpreted as hypothesis-generating.*

**Supplementary Table S2. Exact per-protocol denominators within each risk stratum — baseline-only sensitivity analysis (strata defined by a logistic-regression model using pre-treatment variables only).**

| **Risk stratum** | **Protocol** | **Clinical pregnancies / cycles (n)** | **Rate (%)** |
| --- | --- | --- | --- |
| High probability | Long-acting GnRH agonist (Fresh) | 30/40 | 75.0 |
| High probability | GnRHa-HRT (Frozen) | 22/43 | 51.2 |
| High probability | GnRH antagonist (Fresh) | 15/30 | 50.0 |
| High probability | NC (Frozen) | 15/33 | 45.5 |
| High probability | HRT (Frozen) | 7/30 | 23.3 |
| Low probability | Long-acting GnRH agonist (Fresh) | 7/21 | 33.3 |
| Low probability | NC (Frozen) | 5/20 | 25.0 |
| Low probability | GnRHa-HRT (Frozen) | 8/34 | 23.5 |
| Low probability | GnRH antagonist (Fresh) | 10/43 | 23.3 |
| Low probability | HRT (Frozen) | 13/61 | 21.3 |

*Strata were re-derived from a logistic-regression model fitted on 22 pre-treatment variables only (no estradiol, oocytes retrieved, embryo quality or other post-stimulation/embryo variables). The headline association (long-acting GnRH-agonist fresh transfer vs HRT frozen transfer in the high-probability stratum) persisted (FDR-adjusted p=0.002).*

**Supplementary Table S3. Final hyperparameters for each algorithm (library defaults; no hyperparameter search was performed).**

| **Algorithm** | **Hyperparameter** | **Value** |
| --- | --- | --- |
| Logistic Regression | penalty | l2 |
|  | C | 1.0 |
|  | solver | liblinear |
|  | max_iter | 1000 |
|  | class_weight | None |
|  | random_state | 654321 |
| Random Forest | n_estimators | 100 |
|  | criterion | gini |
|  | max_depth | None |
|  | min_samples_split | 2 |
|  | min_samples_leaf | 1 |
|  | max_features | sqrt |
|  | bootstrap | True |
|  | random_state | 654321 |
| Gradient Boosting | loss | log_loss |
|  | learning_rate | 0.1 |
|  | n_estimators | 100 |
|  | subsample | 1.0 |
|  | max_depth | 3 |
|  | min_samples_split | 2 |
|  | random_state | 654321 |
| XGBoost | objective | binary:logistic |
|  | n_estimators | 100 (library default) |
|  | max_depth | 6 (library default) |
|  | learning_rate | 0.3 (library default) |
|  | subsample | None |
|  | colsample_bytree | None |
|  | eval_metric | logloss |
|  | random_state | 654321 |
| CatBoost | iterations | 1000 (library default) |
|  | learning_rate | auto (library default) |
|  | depth | 6 (library default) |
|  | l2_leaf_reg | 3 (library default) |
|  | silent | True |
|  | random_state | 654321 |
| Extra Trees | n_estimators | 100 |
|  | criterion | gini |
|  | max_depth | None |
|  | min_samples_split | 2 |
|  | min_samples_leaf | 1 |
|  | max_features | sqrt |
|  | bootstrap | False |
|  | random_state | 654321 |

*All models were fitted at documented library default hyperparameters (scikit-learn 1.9.0; XGBoost 3.2.0; CatBoost 1.2.10) under a fixed random seed (654321); no grid or random hyperparameter search was performed. Where a value is shown as "library default", the algorithm uses its internal default when the parameter is unset. The complete, machine-readable parameter dictionaries are available in the reproducibility file (R1_meta.json).*

**Supplementary Table S4. Comparison of the present manuscript with the companion live-birth study (Liu et al., Front Endocrinol 2026;17:1832301), based on an overlapping single-centre DOR cohort: the two studies differ in primary endpoint (clinical pregnancy vs live birth), research question (risk-stratified protocol comparison vs stage-specific prediction), primary model (logistic regression vs CatBoost), and conclusions.**

| **Aspect** | **Present study (this manuscript)** | **Companion study (Liu et al., Front Endocrinol 2026;17:1832301; ref 19)** |
| --- | --- | --- |
| Primary endpoint | Cumulative clinical pregnancy per oocyte-retrieval cycle (event rate 37.3%) | Cumulative live birth (≥ 1 live-born infant at ≥ 28 weeks) per oocyte-retrieval cycle (event rate 22.8%) |
| Primary research question | Hypothesis-generating, risk-stratified comparison of ovarian-stimulation and embryo-transfer protocols within model-defined baseline-prognosis strata | Stage-specific prediction of cumulative live birth at three decision junctures (baseline / post-stimulation / pre-transfer), quantifying the incremental value of information available at each stage |
| Cohort | Largely overlapping single-centre DOR cohort (AMH ≤ 1.1 ng/mL), 2019–2024; n = 1,251 retrieval cycles (train 875 / test 376); analytical unit = oocyte-retrieval cycle | Largely overlapping single-centre DOR cohort (same source population); n = 1,234 retrieval cycles (train 863 / test 371); the small difference in n reflects endpoint-specific complete-case filtering, not a different patient population |
| Predictor set | 37 candidate predictors → random-forest Top-8; SHAP highlights five (embryo quality, female age, male age, estradiol, AMH) | Stage-specific predictor sets; baseline model uses five (female age, male age, AMH, BMI, infertility duration); embryological parameters added at pre-transfer |
| Modelling framework | Six classifiers compared (logistic regression, random forest, gradient boosting, XGBoost, CatBoost, extra trees); the parsimonious logistic regression was adopted as primary on the basis of equivalent discrimination (DeLong p>0.10) and superior calibration (test AUC 0.739) | Six tree-based algorithms compared (decision tree, LightGBM, gradient boosting, XGBoost, CatBoost, AdaBoost) with explicit class-imbalance mitigation; CatBoost selected as primary (pre-transfer test AUC 0.793). The two algorithm panels overlap in only three of six members (gradient boosting, XGBoost, CatBoost); the present study uniquely includes a logistic-regression primary model |
| Treatment-effect analysis | Yes — risk-stratified, exploratory protocol comparison with FDR + Bonferroni correction, explicit collider/confounding caveats, and a baseline-only sensitivity analysis | Not applicable (focus on prediction only; no protocol or treatment-effect comparison) |
| Validation | 70:30 cycle-level held-out split (cycle-level internal validation); 200-split stability; out-of-time validation 2019–2022 vs 2023–2024 (AUC 0.726) | 70:30 cycle-level held-out split; stage-specific internal validation |
| Headline conclusion | Hypothesis-generating: a single protocol-related association survived correction (long-acting GnRH-agonist fresh transfer vs HRT frozen transfer in the favourable-prognosis stratum); requires prospective external validation before any clinical use | Embryological parameters substantially enhance cumulative live-birth prediction whereas ovarian-response markers add little; explicit class-imbalance mitigation and interpretability are essential |
